# Inflammasome activation occurs in CD4^+^ and CD8^+^ T cells during graft-versus-host disease

**DOI:** 10.1038/s41419-023-06138-8

**Published:** 2023-09-25

**Authors:** Sarah Talley, David J. Rademacher, Edward M. Campbell

**Affiliations:** 1 Department of Microbiology and Immunology, Stritch School of Medicine, Loyola University Chicago, Maywood, IL USA; 2 Core Imaging Facility and Department of Microbiology and Immunology, Loyola University of Chicago, Maywood, IL USA

**Keywords:** Inflammasome, Allotransplantation

## Abstract

A severe complication of hematopoietic stem cell transplantation is graft-versus-host disease (GvHD), a reaction that occurs following the transfer of donor immune cells (the graft) into an allogeneic host. Transplanted cells recognize host alloantigens as foreign, resulting in the activation of donor T cells and migration of these pathological cells into host tissues. In this study, we found that caspase-1 is activated in alloreactive murine and human CD4^+^ and CD8^+^ T cells early during acute GvHD (aGvHD). The presence of inflammasome-bound active caspase-1 (p33) and ASC-speck formation confirmed inflammasome activation in these cells. We further measured gasdermin D (GSDMD) cleavage and IL-18 secretion from alloreactive T cells ex vivo. Isolated T cells with high levels of active caspase-1 had a strong inflammatory transcriptional signature and a metabolic phenotype similar to inflammatory myeloid cells, including the upregulation of proinflammatory cytokines and metabolic switch from oxidative phosphorylation to aerobic glycolysis. We also observed oxidative stress, mitochondrial dysfunction, and cell death phenotypes consistent with inflammatory cell death in alloreactive T cells. For the first time, this study characterizes caspase-1 activation in transplanted T cells during aGvHD, using mouse and human models, adding to a body of literature supporting inflammasome function in cells of the adaptive immune system.

## Introduction

Inflammasomes are multiprotein assemblies activated in numerous cell types in response to pathogen derived and sterile inflammatory stimuli, canonically leading to the activation of inflammatory caspases, such as caspase-1, and the release of IL-1β and IL-18. Inflammatory caspases also cleave GSDMD, which forms pores in the plasma membrane that serve as conduits for cytokine release and mediate an inflammatory form of cell death called pyroptosis. While these events are well characterized in innate immune cells, a number of recent studies have found evidence of inflammasome activation in cells of the adaptive immune system, particularly in T cells in the context of pathogen infection and autoimmune diseases. NLRP3 and ASC are known to drive caspase-1 or caspase-8 activation, as well as IL-1β maturation in Th1 and Th17 cells [1, 2]. More recently, CARD8 inflammasome activation in resting human CD4^+^ and CD8^+^ T cells was shown to trigger pyroptosis without detectable IL-1β and IL-18 secretion [3, 4]. CARD8 has since been identified as a sensor HIV-1 protease, driving caspase-1 activation and cell death in CD4^+^ T cells [5]. Quiescent, lymphoid-resident CD4^+^ T cells were also found to undergo caspase-1 dependent pyroptosis following recognition of abortive HIV-1 transcripts by IFI16 [6]. Others have observed NLRP3 inflammasome activation in circulating CD4^+^ T cells and pyroptosis in lymph nodes in viremic HIV-1-infected patients [7]. These studies provide solid evidence that inflammasome activation can occur in CD4^+^ T cells, promoting effector function, inflammatory cytokine secretion and pyroptosis. Whether these cells always commit to pyroptosis or if cytokine secretion occurs from viable T cells, remains unknown [8]. Furthermore, inflammasome activation in CD8^+^ T cells is not well characterized.

In addition to these conventional roles for inflammasomes, some T cell intrinsic, inflammasome-independent functions have been ascribed to inflammasome-forming proteins. One such inflammasome-independent function for ASC was demonstrated in a mouse model of GvHD, where transfer of ASC-deficient T cells conferred protection from disease, while NLRP3-deficient or caspase-1/11-deficient T cells did not improve GvHD outcomes [9]. However, many of these studies fail to account for the existence of numerous inflammasome receptors, many of which are poorly characterized, as well as the redundant roles of caspases as inflammasome effectors. Our previous results suggested that inflammatory caspase activation occurs in donor splenocytes in a model of aGvHD [10, 11]. Here, we demonstrate activation of caspase-1 in alloreactive T cells using mouse and human models of aGvHD. Furthermore, we demonstrate inflammasome activation and pyroptosis in these cells, as well as increased inflammatory transcript expression and metabolic perturbations in cells where caspase-1 is active. These data indicate that caspase-1 activation occurs in pathological alloreactive T cells during GvHD.

## Materials and methods

### Mice

C57Bl/6J (strain #000664), BALB/cJ (strain #000651), GSDMD KO (strain #032663) [12], ASC-citrine (strain #030744) [13] mice and NSG HLA-A2 mice (strain #014570) were obtained from Jackson Laboratory. IQAD Tg mice [10] and were bred in-house. Donor and recipient mice were 6–8 weeks of age. No randomization was used and investigators were not blinded. All mice were maintained in pathogen-free conditions at Loyola University Chicago. All experiments were performed in accordance with protocols approved by Loyola University Chicago’s Institutional Animal Care and Use Committee.

### Induction of GvHD

Splenocytes were harvested from the indicated donor mouse strain and T cells were isolated by negative selection using a negative isolation kit (EasySep Mouse T Cell Isolation kit; STEMCELL Technologies) or a cocktail of biotinylated antibodies (anti-B220 [RA3-6B2], anti-CD11b [M1/70], anti-CD11c [N418]), followed by incubation with magnetic nanoparticles conjugated to streptavidin (BD IMag Streptavidin Particles Plus; BD Biosciences) and magnetic isolation. Purity (>90%) was confirmed by flow cytometry staining for T cells (anti-CD3) (Fig. S1). 0.75–1 × 10^6^ T cells + 1 × 10^7^ BM cells were resuspended in PBS with heparin and intravenously injected into BALB/c recipient mice via tail vein injection. Human HLA-A2 negative PBMCs were isolated from adult healthy donors. 10 million PBMCs were resuspended in PBS with heparin and injected into NSG recipient mice. On the day of transplantation, BABL/c recipients received a total body lethal dose of irradiation (8 Gy) and NSG recipients received (2.5 Gy) from an X-ray source (RS 2000 Biological Research Irradiator). Body weights were recorded and clinical signs observed throughout the experiment. On day 7 (BALB/c) or day 10 (NSG), mice were sacrificed for ex vivo analyses. For survival experiments, experiments were carried out for 80 days. When mice reached weight loss >25% of their initial body weight or hit end of life criteria, mice were considered clinically dead.

### IVIS measurements

Mice were injected i.p. with 150 mg/kg VivoGlo Luciferin (Promega). Mice were anesthetized with 2% isoflurane/air mixture and imaged using the IVIS 100 Imaging system (Xenogen) 10 min following administration of the luciferase substrate. Tissues were extracted from sacrificed mice and imaged. Bioluminescent images were acquired and analyzed using Living Image software (PerkinElmer).

### Western blot

Splenocytes were isolated from control mice or mice with GvHD. Briefly, spleens were passed through a 70 um strainer in with media. Collected splenocytes were cleared of RBCs using ACK lysis buffer (Gibco). Donor T cells were isolated from BALB/c GvHD mice by positive selection using a biotinylated anti-CD3 (145-2C11) antibody or by negative isolation using a negative isolation kit as above (STEMCELL Technologies). For positive selection, magnetic streptavidin particles followed by magnetic isolation was performed as described above. Cells were immediately lysed in in NP-40 lysis buffer containing a protease inhibitor cocktail (Roche). Lysates were collected following 30 min incubation on ice, and protein concentration was quantified by BCA (Pierce; Thermo Fisher Scientific). Samples were mixed with 2× Laemmli sample buffer and boiled for 5 min at 95 °C. Equal amounts of protein were loaded into a 4–15% gradient gel (Bio-Rad) and transferred onto a nitrocellulose membrane (Bio-Rad). The following antibodies were used for WB: anti-mouse caspase-1 (Adipogen), anti-mouse caspase-3 (Cell Signaling Technology), anti-mouse GSDMD (Cell Signaling Technology), anti-β-Actin (Santa-Cruz Biotechnology), and anti-mouse IgG or anti-rabbit IgG conjugated to HRP (Thermo Fisher Scientific). Chemiluminescence was measured using SuperSignal West Femto Chemiluminescent Substrate (Thermo Fisher Scientific) and a FluorChem E machine (Protein Simple).

### Flow cytometry

Splenocytes were isolated as described above. PBMCs and T cells from MLR cultures were isolated using a negative selection human T cell isolation kit (EasySep, STEMCELL technologies). T cells were incubated with the FAM-FLICA Caspase-1 probe following the manufacturer’s protocol (Immunochemistry). 1x probe was added to cells in culture media and incubated for 1 h at 37 °C. Cells were washed in media. In some experiments, cells were additionally labeled with CellRox Red (1:1000, 25 min), MitoSox (1:1000, 15 min), or TMRM (1:1500, 20 min) at 37 °C, following the manufacturer’s instructions. For live/dead staining, cells were washed in PBS and subsequently labeled with Zombie Yellow (BioLegend) 1:1000 in PBS for 20 min. Cells were washed and incubated with Fc Receptor Block (mouse; human) and the following antibodies: anti-mouse CD3 (clone 17A2), anti-mouse CD4 (clone GK1.5, clone RM4-5), anti-mouse CD8 (clone 53-6.7), anti-mouse CD25 (clone PC61), anti-mouse CD44 (clone IM7), anti-mouse CD71 (clone RI7217), anti-human CD45 (clone 2D1), anti-human CD3 (clone UCHT1), anti-human CD4 (clone A161A1), anti-human CD8 (clone SK1) (BioLegend). For Imagestream analysis, cells were subsequently fixed and stained with Hoechst. Samples were measured on an LSRFortessa (BD Biosciences) or Imagestream (Amnis) and data were analyzed using FlowJo or IDEAS (Amnis) software. t-SNE plots were generated using FlowJo.

### RNA isolation, qRT-PCR and RNA sequencing

Splenocytes were isolated in media containing DNAse, 1 mM MgCl_2,_ and FBS, and stained as described above. Cells were sorted by using an LSR Fortessa (Becton Dickinson) using the gating strategy depicted in Supplement 4. Collected cells were centrifuged and lysed in RNA lysis buffer and RNA was isolated using the NucleoSpin RNA isolation kit (Macherey-Nagel). Total RNA concentration and purity were assessed using a Nanodrop spectrophotometer. Isolated RNA was submitted to Novogene for oligo-dT mRNA library preparation and sequencing (NovaSeq PE150) or converted to cDNA using the GoScript Reverse Transcription System (Promega). qRT-PCR was performed using iTaq Universal SYBR Green master mix (SYBR) following the manufacturer’s instructions. The following primers were used: Actin fwd 5′-GTGACGTTGACATCCGTAAAGA-3′, Actin rev 5′-GCCGGACTCATCGTACTCC-3′; IL-6 fwd 5′-CTTCACAAGTCGGAGGCTTAAT-3′, IL-6 rev 5′-ACTCCAGGTAGCTATGGTACTC-3′; caspase-1 fwd 5′-ACAAGGCACGGGACCTATG-3′ caspase-1 rev 5′TCCCAGTCAGTCCTGGAAATG-3′; IL-1β fwd 5′-ATCAACCAACAAGTGATATTCTCCAT-3′, IL-1β rev 5’GGGTGTGCCGTCTTTCATTAC-3’; Nlrp3 fwd 5′-AGCCAGAGTGGAATGACACG-3′, Nlrp3 rev 5′-CGTGTAGCGACTGTTGAGGT-3′, mt-ND4 fwd 5′-AACGGATCCACAGCCGTA-3′, mt-ND4 rev 5′-AGTCCTCGGGCCATGATT-3′; mt-Dloop1 fwd 5′-AATCTACCATCCTCCGTGAAACC-3′, mt-Dloop1 rev 5′-TCAGTTTAGCTACCCCCAAGTTTAA-3′; mt-CytB fwd 5′-GCTTTCCACTTCATCTTACCATTTA-3′, mt-CytB rev 5′-TGTTGGGTTGTTTGATCCTG-3′.

### Immunofluorescence imaging

T cells were isolated from mice with aGvHD by negative selection (EasySep Mouse T Cell Isolation kit; STEMCELL Technologies). Isolated T cells were incubated with the FAM-FLICA Caspase-1 probe as above. Cells were washed, fixed, and incubated with an anti-dsDNA antibody (Abcam) in block with 0.1% saponin for 1 h, followed by incubation with the indicated secondary antibody and Hoechst. Stained T cells were spinoculated onto poly-L-lysine-treated coverslips for 15 min and mounted onto glass slides for imaging with a DeltaVision wide field fluorescence microscope (Applied Precision, GE) equipped with a digital camera (CoolSNAP HQ; Photometrics) and a 1.4-numerical aperture 100× objective lens. Z-stack images were collected and deconvolved with SoftWoRx deconvolution software (Applied Precision, GE). 20 images were acquired per group (day 0 control vs. day 7 GvHD) and the percentage of cells with dsDNA staining relative to total cells per image (Hoechst) was quantified by a blinded investigator using Imaris (Bitplane).

### Subcellular fractionation and cytosolic mtDNA quantification

CD3^+^ T cells were isolated from GvHD or control mice by positive selection as described above. Cytosolic fractions were isolated as described previously [14]. Briefly, one aliquot of isolated cells was resuspended in digitonin buffer (with PIC for protein isolation, without PIC for DNA isolation) and incubated on a rotator for 10 min at 4 °C to allow for plasma membrane permeabilization. Supernatants containing cytosolic fractions were collected following two rounds of centrifugation. The second aliquot of isolated cells was resuspended in NaOH solution and boiled to isolate total cellular mtDNA. Purity of cytosolic fractions was assessed by western blot, using anti-AKT (9272S, Cell Signaling Technology), anti-Bax (sc-7480, Santa Cruz Biotechnology), and anti-GAPDH (sc-365062, Santa Cruz Biotechnology) antibodies. For DNA isolation, proteinase K and buffer AL were added to the isolated cytosolic fractions or total lysates and incubated at 56 °C for 10 min. DNA was isolated using the DNeasy Blood & Tissue Kit (Qiagen) following the manufacturer’s instructions. qRT-PCR was performed as above and cytosolic mtDNA was calculated by the 2^ΔΔCt method, normalizing to total cellular mtDNA for each sample and expressing the data relative to control samples [14].

### Seahorse

FACS sorted cells were centrifuged, resuspended at 100,000 cells/180 μL of Seahorse XF Base Medium, and immediately plated in a 96 well Seahorse XF cell culture microplate. Cells were incubated at 37 °C for 30 min in a non-CO_2_ incubator. The Seahorse XF Real-Time ATP Rate Assay (Agilent) was performed in accordance to the manufacturer’s instructions. Following the assay, cells were lysed and protein concentration was calculated by BCA (Pierce; Thermo Fisher Scientific). Normalized glycolytic and mitochondrial ATP production, basal OCR and ECAR measurements were calculated using Wave software (Agilent).

### Transmission electron microscopy (TEM)

FACS sorted T cells were washed in phosphate buffered saline (PBS) then immersed in PBS containing 4% paraformaldehyde (Electron Microscopy Sciences) and 4% glutaraldehyde (Electron Microscopy Sciences) for 1 h at room temperature. After extensive washing with deionized water, the samples were fixed with deionized water containing 1% osmium tetroxide and 1.5% potassium ferricyanide (Electron Microscopy Sciences) for 1 h at room temperature in the dark. Samples were dehydrated by incubation in an ascending series of alcohols (25, 50, 75, 95, 100%, Electron Microscopy Sciences) followed by incubation in propylene oxide. Next, the samples were incubated in a 1 to 1 ratio of propylene oxide to epoxy resin, comprised of a mixture of EMbed 812, nadic methyl anhydride, dodecenyl succinic anhydride, and 2,4,6-Tris(dimethylaminomethyl)phenol, (Electron Microscopy Sciences), for 12 h on a rotary mixer (Ted Pella, Inc.). The samples were incubated with 100% epoxy resin for 12 h at room temperature on a rotary mixer (Ted Pella, Inc). The epoxy resin was changed and the samples were incubated in epoxy resin for 2 h at room temperature on a rotary mixer (Ted Pella, Inc). The epoxy resin was allowed to polymerize at 70 °C for 36 h then the samples were allowed to cool to room temperature. Ultrathin sections (70 nm) were cut with an ultramicrotome (EM UC7, Leica Microsystems), mounted on formvar- and carbon-coated 200 mesh copper grids, then stained with filtered 1% uranyl acetate and Reynold’s lead citrate prior to imaging. Samples were imaged with a Philips CM 120 transmission electron microscope (TSS Microscopy) equipped with a BioSprint 16 megapixel digital camera (Advanced Microscopy Techniques) by a blinded investigator.

### In vitro T cell activation, LDH, and ELISAs

Donor T cells were extracted from spleens of mice with aGvHD (day 7 post transplant) or control C57Bl/6 mice by negative isolation (STEMCELL Technologies) as described above. Cells were plated in media at 4 × 10^5^ cells/well in 96 well round-bottom tissue culture plates. After 4 h, supernatant was collected and used for LDH or cytokine quantification. LDH was measured using the CyQUANT^TM^ LDH Cytotoxicity Assay Kit (Invitrogen) and IL-1β and IL-18 levels were measured by ELISA (DuoSet Mouse ELISAs, R&D Systems). Mouse T cells isolated from C57Bl/6 mice were activated using Dynabeads Mouse T-Activator CD3/CD28 (Gibco), according to the manufacturer’s protocol, in media with IL-2. After 48 h, cells were collected and lysed for western blot analysis of caspase-1 expression.

### Mixed lymphocyte reaction (MLR)

Monocytes were isolated from PBMCs using the EasySep Human Monocyte Isolation Kit (STEMCELL Technologies) and differentiated with 20 ng/mL GM-CSF and IL-4 for 7 days. T cells were isolated from PBMCs using the EasySep Human T Cell Isolation Kit (STEMCELL Technologies) and mixed with DCs 5:1. In some experiments, T cells were labeled with 5 μM CellTrace Violet (CTV) (Life Technologies) following the manufacturer’s instructions. 4 and 7 days later, cells from MLR cultures were harvested and analyzed by flow cytometry as described above. Proliferation was assessed by calculating the loss of CTV relative to CTV signal measured on day 0. For microscopic analyses, T cells were isolated from MLR cultures by negative selection as above and re-plated.

### Microscopy and quantification of PI-positive cells

T cells isolated from PBMCs, MLR cultures, or Vbp-stimulated T cells were incubated with PI (Immunochemistry, 1:333) for 1 h in 24-well tissue culture plates. Images were acquired using a Nikon Ti2 inverted microscope equipped with a 20×/0.8 NA objective. Data were analyzed by a blinded investigator using an algorithm generated in FIJI. Specifically, masks were built around each cell in the bright-field image and PI channel to enumerate the total number of cells and the number of PI-positive cells, respectively. This algorithm was applied to all images acquired and the fraction of PI-positive cells/total cells was then calculated for each image.

### Statistical analysis

Data from ≥3 independent experiments are depicted in dot blots ± SEM, where each dot represents an individual mouse, or bar graphs represented as the mean ± SEM. Sample sizes were chosen based on technical feasibility and the number of independent experiments required for statistical significance. Statistical significance was determined using a student’s *t* test when comparing two groups or one-way ANOVA followed by a Bonferroni multiple comparisons test when comparing multiple groups using GraphPad Prism software (GraphPad Software, Inc.). RNA sequencing data and significance were analyzed using R.

## Results

### Caspase-1 is active in allogeneic CD4^+^ and CD8^+^ T cells in mice with aGvHD

**Fig. 1:**
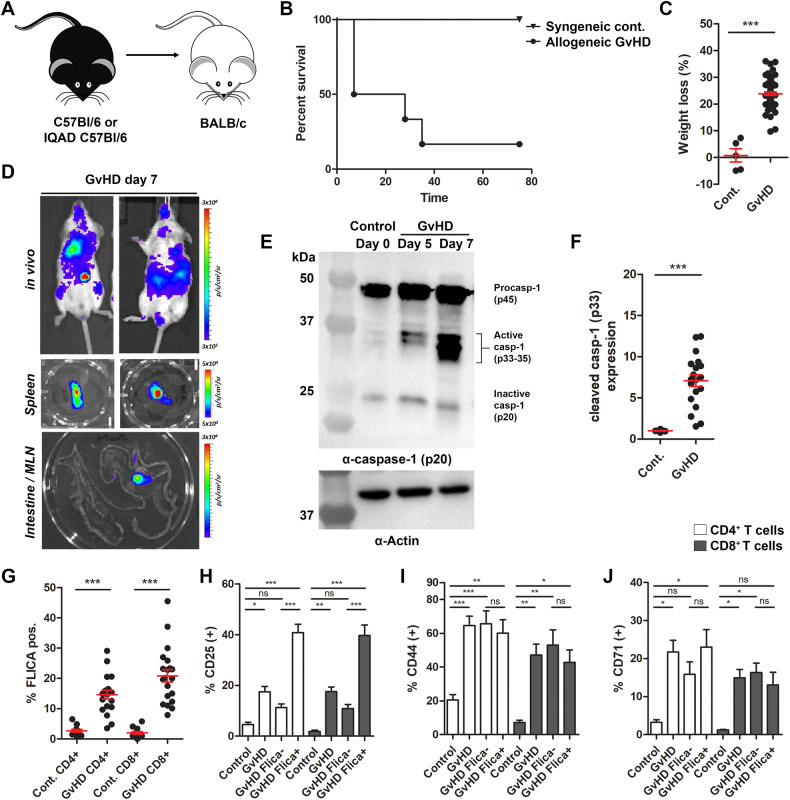
Caspase-1 is activated in donor allogeneic T cells during aGvHD. **A** 1 × 10^6^ T cells + 1 × 10^7^ BM cells from IQAD C57Bl/6 Tg or WT C57Bl/6 mice were transferred into irradiated 5–6 week old BALB/c recipients to induce GvHD. **B** Survival was measured over the course of disease, with mice losing >25% of their initial body weight marked as clinically dead. **C** Percent change in body weight on day 7 in mice that received syngeneic (control) or allogeneic T cell transplants. **D** Representative images of mice with aGvHD on day 7 following transplantation of IQAD biosensor T cells. IVIS images were acquired in vivo and ex vivo in lymphoid organs. Scale bars: in vivo 3e3–3e4, spleens 5e3–5e4, and MLN 3e3–3e4 radiance (p/s/cm2/sr). **E** On day 5 or 7, spleens were harvested from mice with aGvHD or control mice (day 0), and T cells were isolated by negative selection and lysed for WB analysis of caspase-1 expression. **F** Quantification of caspase-1 p33 expression in T cells isolated on day 7 in mice with aGvHD. **G** Splenocytes isolated from control and aGvHD mice were incubated with the FAM-FLICA Caspase-1 probe, stained for T cell surface markers (α-CD3, α-CD4, and α-CD8), and caspase-1 activation (% Flica-positive) was quantified by flow cytometry. **H**–**J** Expression of T cell activation markers, CD25, CD44 and CD71 was measured in total (GvHD), Flica-positive (GvHD Flica+), or Flica-negative (GvHD Flica-) CD4^+^ and CD8^+^ T cells from mice with aGvHD and control mice (gated on single cells, CD3^+^; see gating strategy in Supplement 1). Data presented are averages ± SEM from >3 independent experiments. **C**, **F** Student’s *t* test or **G**–**J** one-way ANOVA and Bonferroni post hoc test, *p* < 0.05 was considered significant.

A number of groups have observed irradiation-induced inflammasome/inflammatory caspase activation in recipient tissues in mice [15–17] and in patients with severe GvHD [15], although there has been little investigation into the contribution of inflammatory caspase activation in alloreactive T cells, the cells driving pathology in this disease. Our previous data suggested that inflammatory caspases were active in allogeneic splenocytes [10, 11]. To interrogate this further, we used the C57Bl/6 (H-2Kb) → BALB/c (H-2Kd) MHC major mismatched model of aGvHD (Fig. 1A). This model results in robust engraftment and clinical manifestations of aGvHD pathology in allogeneic recipient mice [10, 11, 18, 19]. 0.75–1 × 10^6^ T cells and 1 × 10^7^ bone marrow cells from C57Bl/6 mice were transplanted into lethally irradiated BALB/c recipient animals (Fig. S1A, B) and disease progression was measured. We detected two waves of acute disease, the first occurring one week post transplantation. At this time point, 50% of the mice were moribund and had lost >25% of their initial body weight (Fig. 1B, C). By transferring T cells expressing a bioluminescent reporter activated by active caspase-1, we were able to track this population of donor T cells in mice with aGvHD [10, 11]. Bioluminescence was detected on day 7 in vivo and ex vivo, predominantly in the spleen and lymph nodes (Fig. 1D). To more rigorously assess caspase-1 activation in donor T cells, we harvested splenocytes from control mice or aGvHD mice. T cells were isolated from total splenocytes and the proteolytic cleavage of caspase-1 was measured by western blot. We detected increased expression of active caspase-1 by day 7 post transplant, as measured by the appearance of bands ~33–35 kDa (Fig. 1E, F), consistent with the size corresponding to the active species of caspase-1 observed when it is bound to an inflammasome [20]. We were unable to detect activation of caspase-1 in α-CD3/CD28-stimulated T cells in vitro (Fig. S2), suggesting that this response is specific to alloreactive T cells.

### Caspase-1 is active in alloreactive T cells

We next turned to flow cytometry to determine the T cell population in which caspase-1 was active. The FAM-FLICA caspase-1 probe was applied to splenocytes from control or GvHD mice, and cells were subsequently stained with antibodies for T cell surface and activation markers. Active caspase-1 was detected in both CD4^+^ and CD8^+^ donor T cells isolated from mice with GvHD (Figs. 1G and S1C). To assess if caspase-1 activation occurred in the alloreactive cells, we quantified expression of CD25, CD44, and CD71 in Flica-positive and negative CD4^+^ and CD8^+^ T cells [21, 22]. As expected, we measured an increased percentage of all activation markers in CD4^+^ and CD8^+^ T cells from GvHD mice relative to control (Fig. 1H–J). While all activation markers were enriched in Flica-positive cells, indicating that this population contains pathogenic alloreactive T cells, we also measured increased expression of CD44 and CD71 in T cells that were Flica-negative (Fig. 1H–J). Alloreactive T cell subsets were further visualized using t-distributed stochastic neighbor embedding (t-SNE) (Fig. S3). There was a distinct shift in T cells in GvHD mice compared to control, specifically an enrichment in clusters that had high levels of active caspase-1 (Fig. S3B, C). In the GvHD t-SNE plots, nearly all cells in clusters with the highest Flica signal (gates 1 and 2) contained alloreactive T cells that were positive for CD25 and CD44 (Fig. S3D–F). Expression of CD25 and CD44 was reduced in clusters with medium Flica signal (gate 3), and cells within clusters with the lowest Flica intensity (gates 4 and 5) had little-to-no expression of CD25 or CD44 (Fig. S3D–F). Together, these data demonstrate that caspase-1 is active in alloreactive T cells in mice with acute GvHD.

### T cells with active caspase-1 exhibit inflammatory transcriptional signatures

**Fig. 2:**
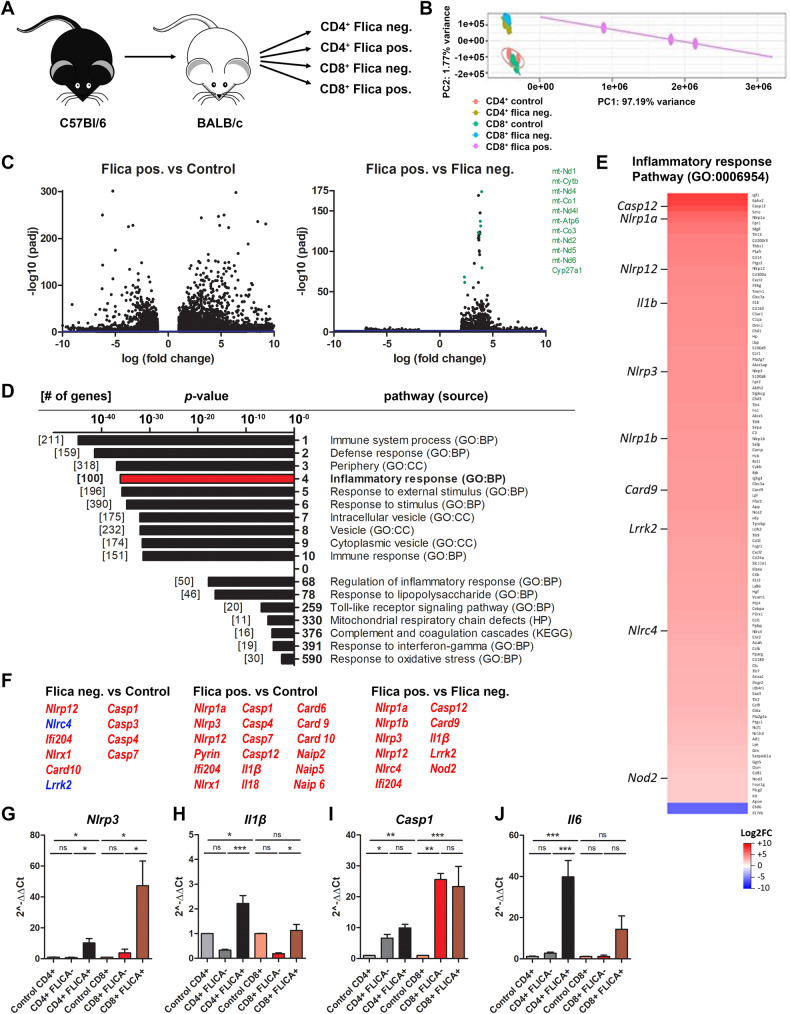
Transcriptional changes in inflammation-associated genes in T cells with active caspase-1. **A** Allogeneic T cells form donor C57Bl/6 mice were transplanted into irradiated BALB/c mice, and on day 7 following T cell transplant, caspase-1 Flica-stained splenocytes from 1–2 control mice and 8–12 mice with aGvHD were sorted into the indicated groups by FACS. Immediately post sort, cells were spun down and RNA extracted for RNA seq (**B**–**F**) or qRT-PCR analysis of inflammatory transcript expression (**G**–**J**). **B** PCA plots of the RNA-seq data depicting clustering of the indicated cell populations. **C** Volcano plots of DEGs *p*_adj_ < 0.05 depicting significance (y-axis) vs. the log fold change (x-axis) from RNA-seq datasets comparing the Flica-positive CD8^+^ T cells to control (left) of Flica-negative (right) populations. **D** Pathway analysis from the RNA-seq dataset comparing Flica-positive vs. Flica-negative CD8^+^ T cell samples. Pathways (and respective source code) are listed numerically in order of significance and the number of significant DEGs within each pathway are bracketed. All DEGs within the Inflammatory Response Pathway, comparing Flica-positive vs Flica-negative CD8^+^ T cells from GvHD mice are shown (**E**) and transcripts relating to inflammasome-forming and associated proteins are listed for all analyses (**F**). qRT-PCR on RNA isolated from sorted Flica-positive, Flica-negative, and control CD4^+^ and CD8^+^ T cells was performed using primers for Nlrp3 (**G**), proIL-1b (**H**), caspase-1 (**I**) and IL-6 (**J**). **G**–**J** Data presented are averages ± SEM from ≥3 independent experiments. One-way ANOVA and Bonferroni post hoc test, *p* < 0.05 was considered significant.

We next sought to phenotypically characterize this population of alloreactive T cells that exhibited inflammasome/caspase-1 activation. To this end, we developed a FACS strategy that allowed us to specifically separate out cells in which caspase-1 was active from those where there was no active caspase-1 (Fig. S4). We collected CD4^+^ and CD8^+^, Flica-positive and Flica-negative T cells from mice 7 days following allogeneic cell transplant (Fig. 2A). To unbiasedly profile transcriptional changes in Flica-positive alloreactive T cells, we first performed RNA sequencing on the sorted Flica-positive and Flica-negative CD8^+^ T cell populations. We did not obtain enough Flica-positive CD4^+^ cells to perform a similar analysis on these cells. As a control, RNA was isolated from sorted Flica-negative control T cells from WT C57Bl/6 mice, since we were unable to sort enough donor T cells from syngeneic recipients for downstream RNA isolation and sequencing. Principal component analysis (PCA) showed clustering of Flica-positive, Flica-negative and control cells, indicating transcriptomic profiles differ between these populations of cells (Fig. 2B). RNA-seq analysis identified thousands of differentially expressed genes (DEGs) in the Flica-positive CD8^+^ T cells relative to control and Flica-negative populations (*p*_adj_ < 0.05) (Fig. 2C). We focused our analysis on the Flica-positive vs. Flica-negative dataset to identify transcriptomic changes that occurred in cells with high caspase-1 activity relative to cells that were in the same microenvironment and exposed to the same stimuli but had undetectable caspase-1 activity. Pathway analysis revealed that the Inflammatory Response pathway was within the top 5 of the most enriched gene ontology (GO) terms (Fig. 2D). DEGs within this pathway included a number of inflammasome-forming receptors and associated proteins, other nod-like receptors (NLRs) and pattern recognition receptors (PRRs), toll-like receptors (TLRs), caspases, proinflammatory cytokines, and damage-associated molecular patterns (DAMPs) (Figs. 2E, F and S5). Transcripts for inflammasome proteins NLRP1a, NLRP1b, NLRP3, NLRP12, IFI204, NLRC4, and Pyrin were all significantly upregulated in the Flica-positive cells, relative to the Flica-negative and/or control CD8^+^ T cells (Figs. 2E, F and S5A). Caspases-1, -3, -4, and -7 were upregulated in both Flica-positive and negative T cells, but there was no significant difference between the Flica-positive and Flica-negative population (Figs. 2E, F and S5B). Several other pathways related to inflammation, LPS and TLR signaling, complement activation, interferon signaling, and mitochondrial deficits and oxidative stress were also enriched in the Flica-positive population (Figs. 2D and S5C). To validate the sequencing data, we performed qRT-PCR on sorted Flica-positive or -negative CD4^+^ and CD8^+^ T cells and quantified expression of select inflammatory transcripts that were identified as significantly upregulated in Flica-positive, CD8^+^ T cells, such as caspase-1, NLRP3 and proIL-1β. We also measured expression of transcripts that were absent from the RNA-seq dataset but would be expected to be upregulated in alloreactive cells, such as IL-6. Nlrp3 and proIL-1β were upregulated in the Flica-positive CD8^+^ T cells relative to the Flica-negative population, while caspase-1 transcript expression was increased in both Flica-positive and negative cells, consistent with the RNA-seq datasets (Fig. 2G–I). IL-6 expression was not significantly different in the sorted CD8^+^ T cell groups, however, there was a robust increase in IL-6 expression in Flica-positive CD4^+^ T cells relative to the control and Flica-negative populations (Fig. 2J). Together, these data demonstrate that Flica-positive T cells have an inflammatory transcriptomic signature.

### Dysfunctional mitochondria, mtROS and mtDNA accumulate in cells with active caspase-1

**Fig. 3:**
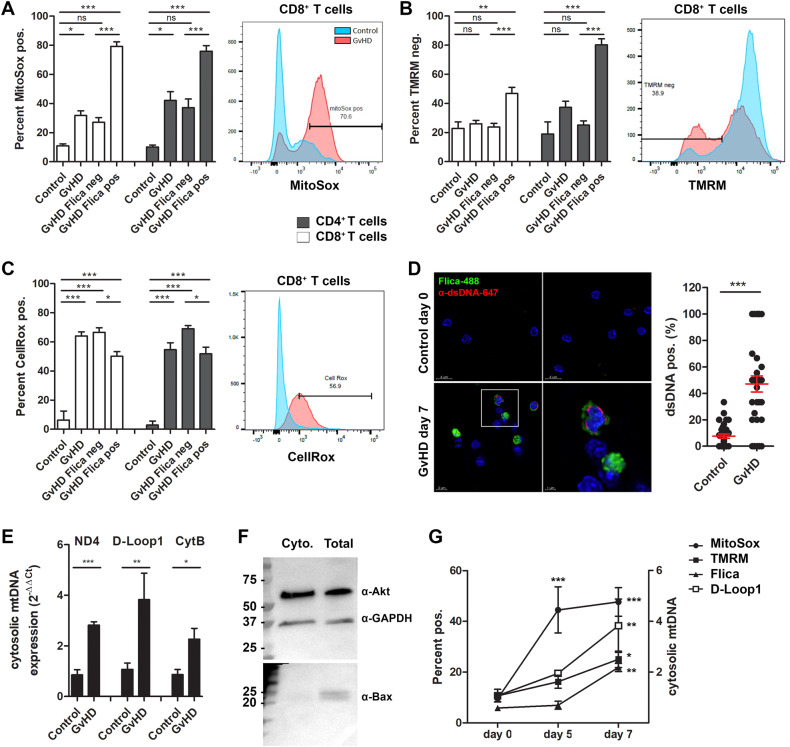
Mitochondrial ROS production and DNA release precede caspase-1 activation in alloreactive T cells. Splenocytes isolated from mice with aGvHD (day 7 post transplant) or control mice were stained with the Flica-caspase-1 probe, followed by MitoSox or TMRM and antibodies for cell surface markers. **A–C** MitoSox, TMRM, and CellRox signal was measured in total (GvHD), Flica-positive (GvHD Flica pos), or Flica-negative (GvHD Flica neg) CD4^+^ and CD8^+^ T cells (gated on single cells, CD3^+^; see gating strategy in Supplement 1). (**A–C**, right) Representative histograms of MitoSox, TMRM, and CellRox staining in CD8^+^ T cells from *n* = 1 control and *n* = 1 GvHD sample are shown. **D–E** T cells were isolated from total splenocytes and cytosolic dsDNA was measured ex vivo. **D** T cells were labeled with Flica and subsequently stained with an anti-dsDNA antibody and dsDNA was measured in Flica-positive cells. The percent of + dsDNA positive cells was quantified relative to the total number of cells (Hoechst) for each image acquired. Equal number of images were acquired for each group (control vs. GvHD) from 3 independent experiments. **E** Cytosolic or total mtDNA was quantified by qRT-PCR using the indicated primers. **F** Representative WB of protein isolated from cytosolic fractions or total lysate, stained with the indicated antibodies. Data are expressed relative to total mtDNA obtained from each sample, normalized to mtDNA detected in control samples. **G** On days 5 and 7 post GvHD induction and MitoSox, TMRM, CellRox, and Flica signal were quantified by flow cytometry or mtDNA was isolated and quantified by qRT-PCR. Data presented are averages ± SEM from ≥3 independent experiments. One-way ANOVA and Bonferroni post hoc test, *p* < 0.05 was considered significant.

Mitochondrial damage leads to the release of mitochondrial-derived DAMPs that directly and indirectly trigger inflammasome activation. Some of the most significant DEGs in Flica-positive, CD8^+^ T cells were transcripts found in the mitochondrial respiratory chain defects, response to oxidative stress, and superoxide metabolic process pathways (Fig. 2C [green]-2D), leading us to hypothesize that mitochondrial dysfunction was an upstream event triggering inflammatory transcript expression and caspase-1 activation in alloreactive T cells. We measured increased mitochondrial ROS (mtROS) in CD4^+^ and CD8^+^ T cells isolated from mice with aGvHD relative to control cells, with most of the mitoSox signal residing in cells with active caspase-1 (Fig. 3A). We also detected a loss in TMRM fluorescence intensity in Flica-positive CD8^+^ T cells (Fig. 3B). Since TMRM accumulates in active mitochondria with intact membrane potentials, this loss in signal indicates that there is increased dysfunctional mitochondria in the CD8^+^ T cell population with ongoing caspase-1 activation. Total cellular levels of oxidative stress were also elevated in alloreactive T cells (Fig. 3C). Damaged mitochondria can release mtDNA into the cytoplasm, which triggers activation of multiple inflammasomes and activation of proinflammatory and innate immune signaling pathways [23]. By day 7 post transplant, we detected increased cytosolic dsDNA in T cells isolated from mice with aGvHD, and the dsDNA signal accumulated in Flica-positive cells (Fig. 3D). We also measured increased mtDNA by qRT-PCR of DNA isolated from cytosolic cellular fractions (Fig. 3E, F). Kinetic experiments further revealed that mtROS generation preceded caspase-1 activation, mtDNA release, and mitochondrial dysfunction (Fig. 3F). Together, these data demonstrate that mitochondrial dysfunction, oxidative stress, and the release of mtROS and mtDNA occur in alloreactive T cells and may trigger inflammatory signaling and inflammasome activation.

### Metabolic changes in alloreactive T cells with active caspase-1

**Fig. 4:**
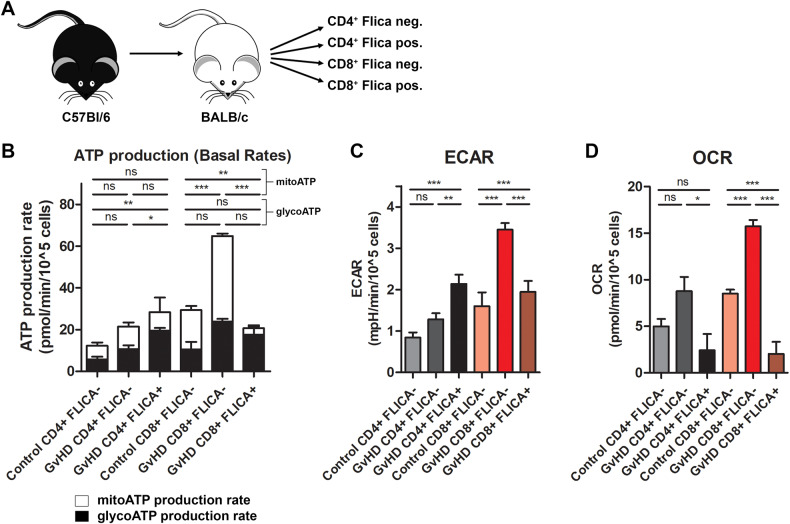
Caspase-1 activation in T cells drives metabolic reprogramming. **A** Allogeneic T cells from donor C57Bl/6 mice were transplanted into irradiated BALB/c mice, and on day 7 following T cell transplant, caspase-1 Flica-stained splenocytes from 1–2 control mice and 8–12 mice with aGvHD were sorted into the indicated groups by FACS. **B–D** Immediately post sort, cell metabolism was analyzed using the Seahorse XF Real-Time ATP Rate Assay (Agilent). **C**, **D** Baseline ECAR and OCR measurements for each group are plotted. Data presented are averages ± SEM from 3 independent experiments. One-way ANOVA and Bonferroni post hoc test, *p* < 0.05 was considered significant.

Alloreactive T cells undergo substantial changes in their metabolic profile, including simultaneous increases in mitochondrial oxidative phosphorylation (OXPHOS) and aerobic glycolysis that help these cells meet increased energy demands needed to elicit their effector function [24]. Dysfunctional mitochondria, mtROS, and inflammasome activation can all influence cellular metabolic function. Specifically, inflammasome activation is also intimately linked to immunometabolism, metabolic reprogramming that occurs in innate and adaptive immune cells upon cell activation. Since inflammatory cells typically shift their metabolism away from OXPHOS and towards glycolysis, we hypothesized that caspase-1 activation in alloreactive T cells may promote a similar metabolic reprogramming. To interrogate the metabolic changes in Flica-positive versus Flica-negative alloreactive T cells, we performed the same FACS strategy as before (Fig. 4A). Sorted cells from control and GvHD mice were plated and metabolic phenotypes were measured using Seahorse technology. Flica-negative CD8^+^ T cells isolated from mice with GvHD were highly energetic, exhibiting increased ATP production rate, oxygen consumption (OCR) and extracellular acidification (ECAR) rates (Fig. 4B–D). OCR and ECAR were also increased in Flica-negative CD4^+^ T cells (Fig. 4C, D). Conversely, while Flica-positive CD4^+^ and CD8^+^ T cell populations had detectable ECAR, mitochondrial ATP production and oxygen consumption were significantly reduced (Fig. 4B–D). These data demonstrate that alloreactive T cells with ongoing inflammasome/caspase-1 activation have a distinct metabolic signature, consistent with metabolic reprogramming characteristic of inflammatory cells.

### Inflammasome/caspase-1 activation in alloreactive T cells triggers GSDMD cleavage, IL-18 production, and morphological changes associated with cell death

**Fig. 5:**
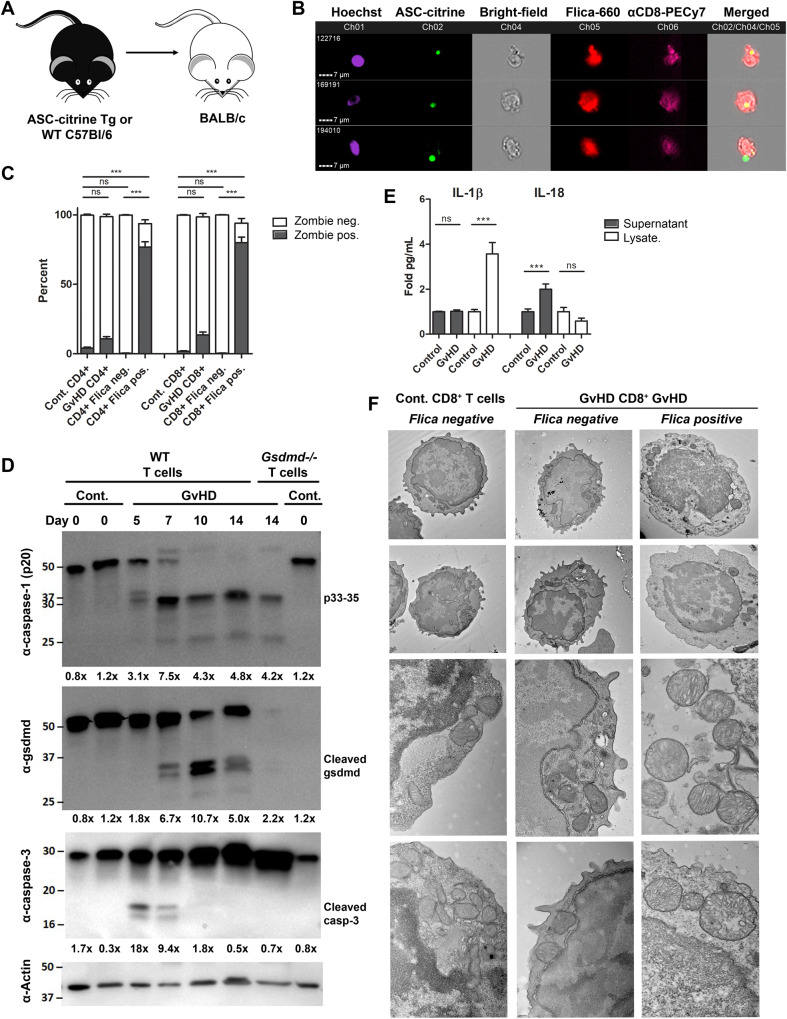
Inflammatory and pyroptotic phenotypes in alloreactive T cells. **A** 0.75–1 × 10^6^ T cells + 1 × 10^7^ BM cells from ASC-citrine expressing C57Bl/6 Tg or WT C57Bl/6 mice were transferred into irradiated 5–6 week old BALB/c recipients to induce GvHD. **B** On day 7 following transplant, splenocytes were harvested from mice with aGvHD or control mice, stained with the FAM-FLICA Caspase-1 probe followed by T cell surface markers and Hoechst and measured by Amnis Imagestream. **B** Representative images of Flica-positive cells with ASC-speck formation. **C** Flica-stained splenocytes were subsequently stained with Zombie yellow dye and Zombie uptake was quantified in total GvHD T cells or in the Flica-negative or Flica-positive populations as in Fig. 1. **D** Western blots were performed on T cell lysates, measuring caspase-1 activation, GSDMD cleavage, and caspase-3 cleavage. Calculations were performed using ImageJ and quantifications are shown below. **E** T cells were isolated from mice with aGVHD (day 7 post transplant) or control mice. Cells were cultured in vitro for 4 h and supernatants and lysates were collected. IL-1ß and IL-18 cytokine levels were quantified by ELISA. **F** Representative TEM images of sorted Flica-positive and Flica-negative CD8+ T cells isolated from control mice or mice with aGvHD. Data in **A**–**D** are representative from >3 independent experiments. Data in **E** are representative from 2 experiments, *n* = 8 mice per experiment. One-way ANOVA and Bonferroni post hoc test, *p* < 0.05 was considered significant.

The increased expression of inflammasome-bound caspase-1 (p33) and upregulation of inflammasome transcripts in alloreactive T cells suggested involvement of an inflammasome. Caspase-1 is recruited into most inflammasome complexes via the adapter molecule ASC. To further examine inflammasome activation in alloreactive T cells, we transplanted T cells expressing an ASC-citrine reporter [13] in which assembled inflammasome complexes can be visualized by the formation of specks (Fig. 5A). We identified some cells with ASC perinuclear speck formation in cells that were also positive for Flica, confirming activation of an inflammasome in alloreactive T cells, albeit ASC specks in T cells were smaller and more sparse than those measured in BMDMs (Figs. 5B and S6A, B). The bright-field images revealed morphological changes in alloreactive T cells, including a ruptured morphology and a ballooning cytoplasm that was not observed in T cells isolated from control mice (Fig. S6C). These morphological features are highly characteristic of activated and dying cells, leading us to hypothesize that this population of alloreactive, Flica-positive T cells was pyroptotic. To test if these cells were dying, splenocytes isolated from control and GvHD mice were incubated with a dye (Zombie yellow) that is permeant to cells with damaged plasma membranes or pores but excluded from living cells or early apoptotic cells with intact membranes. We measured significant Zombie uptake in a majority of Flica-positive, CD4^+^ and CD8^+^ T cells from GvHD mice, while there was little-to-no Zombie uptake in the Flica-negative populations (Fig. 5C). Notably, there was a population of Flica-positive, CD4^+^ and CD8^+^ T cells that remained Zombie negative (~10-20%) (Fig. 5C). Following inflammasome formation, active caspase-1 processes a number of cellular substrates, including GSDMD, which forms pores in the plasma membrane and initiates pyroptosis. If alloreactive T cells are pyroptotic, we would expect to measure GSDMD processing in addition to increased Zombie uptake. We measured increased expression of cleaved GSDMD following the presence of active caspase-1 (days 7–14 post transplant) in alloreactive T cells (Fig. 5D). Although active caspase-3 was also elevated early following GvHD induction, consistent with T cell activation-induced cell death (AICD), caspase-1 activation and cleaved GSDMD persisted after expression of active caspase-3 dissipated (Fig. 5D).

In addition to triggering pyroptosis, active caspase-1 processes proinflammatory cytokines, proIL-1β and proIL-18, into their biologically active form that gets secreted through GSDMD pores. proIL-1β and -IL-18 transcripts were upregulated in Flica-positive cells in our RNA-seq and qRT-PCR analyses (Fig. 2E, F, H), so we next wanted to determine if alloreactive T cells secrete these inflammasome-dependent cytokines. T cells isolated from mice with aGvHD were cultured in vitro for 4 h and IL-1β and IL-18 levels were measured in the lysate and supernatant. Despite significant IL-1β upregulation in T cell lysates, we did not detect IL-1β production from alloreactive T cells, although there was a modest increase in IL-18 secretion and increased Zombie uptake without abundant LDH release(Figs. 5E and S6D–E).

To better characterize alloreactive T cells with active caspase-1, we examined these cells by transmission electron microscopy (TEM). In contrast to control and Flica-negative CD8^+^ T cells, TEM analysis of cells with active caspase-1 showed an electron lucent cytoplasm, dilation of the perinuclear space, and vast changes in mitochondrial ultrastructure (Figs. 5F and S7). Specifically, the mitochondria appeared enlarged and rounded (Figs. 5F and S7). Similar morphological features were observed in CD4^+^ T cells isolated from mice with aGvHD (Fig. S8). We did not observe characteristic morphological changes that occur in cells dying by apoptosis in the Flica-positive alloreactive T cells, such as cell shrinkage, membrane blebbing, nuclear membrane integrity, and chromatin condensation. Together these data demonstrate that multiple caspases are activated and may initiate inflammasome-dependent cytokine secretion and cell death in alloreactive T cells. Most cells with high caspase-1 activity possess distinctive phenotypes of cells dying an inflammatory form of cell death, although not all cells with active caspase-1 are dying, as there is a consistent population of living or pre- or non-pyroptotic (Zombie-negative) alloreactive T cells with active caspase-1 in mice with aGvHD.

### Caspase-1 is activated in alloreactive human T cells

**Fig. 6:**
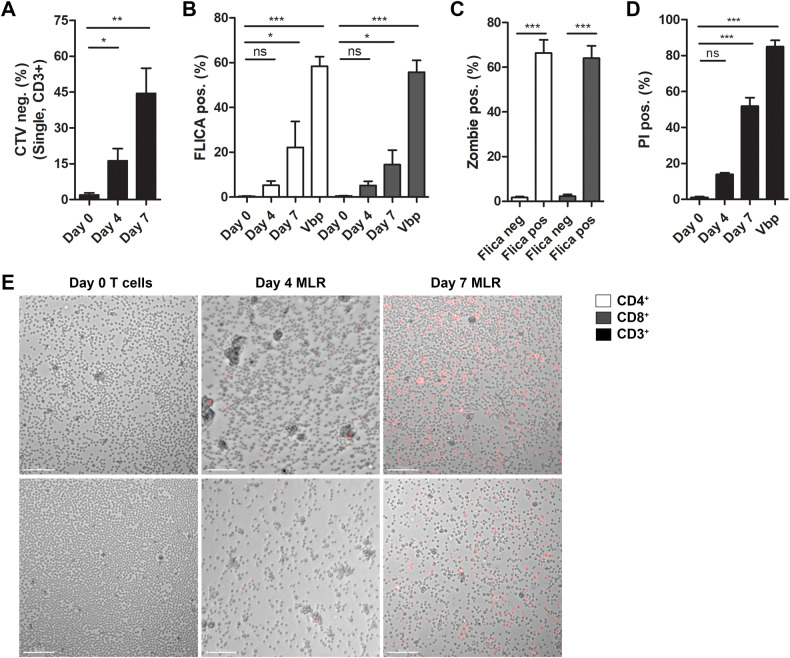
Caspase-1 activation and pyroptosis in human T cells in MLR. T cells isolated from PBMCs were labeled with CTV and mixed with moDC’s (5:1) derived from sex-matched donors. **A**, **B** On day 4 and day 7, cells were labeled with the Flica-caspase-1 probe, Zombie dye, and antibodies to detect T cell surface markers, and the percentages of proliferating cells (CTV-negative) and cells with active caspase-1 (Flica-positive) were quantified by flow cytometry. **C** Zombie uptake was also measured in the Flica-negative and Flica-positive CD4^+^ and CD8^+^ T cells. **D**, **E** T cells were isolated by negative selection from MLR cultures on day 4 or day 7 or from total PBMCs (day 0) and cells were incubated with PI for 1 h, imaged (Nikon Ti2, 20x), and PI-positive cells were quantified (FIJI). As a positive control for caspase-1 activation in these experiments, T cells isolated from PBMCs were stimulated with 20 μM Vbp for 22 h, and **B** Flica signal, **C** Zombie uptake, and **D** PI uptake was measured as described above. Data presented are averages ± SEM from 3 independent experiments using T cells isolated from PBMCs from 3 different donors. Scale bar in **E** = 100μm. One-way ANOVA and Bonferroni post hoc test, *p* < 0.05 was considered significant.

The above data indicate that caspase-1 activation occurs in alloreactive murine T cells in the context of GvHD. To determine if caspase-1 was similarly activated in human T cells, we first used an in vitro mixed lymphocyte reaction (MLR) model to induce T cell alloreactivity, whereby monocyte derived dendritic cells (moDCs) from one donor are mixed with T cells from a different donor. T cells were labeled with CellTrace Violet (CTV) dye to monitor cell proliferation. We measured increased proliferation in T cells by day 7, confirming that a portion of the cells were alloreactive (Fig. 6A). At this time point, we also measured increases in the percentage of cells that were Flica-positive (Fig. 6B). Consistent with the mouse model, Zombie dye uptake was exclusive to the Flica-positive T cells (Fig. 6C). We also visualized propidium iodide (PI) uptake in T cells freshly isolated from peripheral blood mononuclear cells (PBMCs, day 0) or T cells isolated from MLR cultures (days 4 and 7) and measured a significant increase in PI-positive cells by day 7 (Fig. 6D, E), when we were also measuring increased proliferation, activation of caspase-1, and loss of membrane permeability by flow cytometry (Fig. 6A–C). As a positive control for caspase-1-induced pyroptosis in these experiments, T cells were stimulated in vitro with Val-boroPro (Vbp), which triggers CARD8 inflammasome activation and pyroptosis in resting human T cells [3, 4]. Vbp robustly triggered pyroptosis, as measured by the percentage of Flica-positive cells and PI uptake (Fig. 6B, D). These data confirm that caspase-1 is activated in a subset of alloreactive T cells, supporting our observations in the murine GvHD system.

**Fig. 7:**
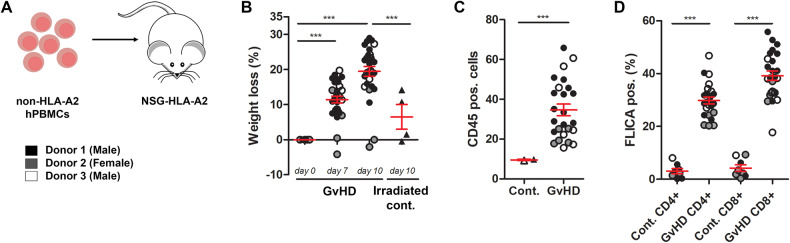
Caspase-1 activation and pyroptosis in human alloreactive T cells during aGvHD. **A** Ten million PBMCs from healthy donors were transferred into sex-matched NSG mice. **B** Body weights were measured over the course of disease in mice that received the PBMC transplant (GvHD) and irradiated control mice that received no PBMCs (Cont.). On day 10, splenocytes were harvested from irradiated control or GvHD mice. **C** Engraftment was measured by quantifying the percentage of CD45-positive human donor cells. **D** Caspase-1 activation was measured in CD45^+^, CD3^+^, CD4^+^ or CD8^+^ T cells by Flica. Data presented are averages ± SEM from 3 independent experiments using T cells isolated from PBMCs from 3 different donors. One-way ANOVA and Bonferroni post hoc test, *p* < 0.05 was considered significant.

We next utilized a humanized mouse model of GvHD where non-HLA-A2 human PBMCs are transplanted into immunodeficient NSG mice engineered to express human HLA-A2 (Figs. 7A and S1D). In this model, donor human T cells respond to allo-MHC and xenoantigens expressed by the recipient, resulting in pathogenesis similar to human aGvHD [25]. 10 days following PBMC transplantation, when over 50% of the mice had reached end of life criteria as measured by weight loss (Fig. 7B), mice were euthanized and caspase-1 activation was quantified in the engrafted human CD4^+^ and CD8^+^ T cell populations. Engraftment was confirmed by measuring the percentage of CD45-positive human cells in mice that received the human PBMC transplant compared to irradiated controls that did not receive any PBMCs (Fig. 7C). As before, we measured increased caspase-1 activation in both CD4^+^ and CD8^+^ T cells (Fig. 7D), suggesting that caspase-1 is similarly activated in alloreactive human T cells.

## Discussion

In this study, we investigated caspase-1 activation in allogeneic T cells in mouse and human models of GvHD. Consistent with our prior studies [10, 11], we detected caspase-1 activation in mice early during aGvHD development (Fig. 1B–G). We specifically measured an increase in a band ~33 kDa (Figs. 1E, 2C, and 6E), which corresponds to the active species of caspase-1 when it is bound to an inflammasome complex [20]. RNA-seq analysis revealed upregulation of multiple transcripts encoding inflammasome-forming proteins in T cells isolated from GvHD mice, including NLRP1, NLRP3, NLRP12, Pyrin, and IFI204 (Fig. 2E–G). We also measured ASC-speck formation in some cells that were also positive for Flica (Fig. 5B). Taken together, these results reveal changes at the protein, transcript, and metabolic level that are defining features of inflammasome activation.

To date, only NLRP3, AIM2, CARD8 and IFI16 inflammasomes have been shown to be activated in T cells. It is unlikely that CARD8 is the inflammasome receptor activated in alloreactive T cells, since rodents do not have a CARD8 homolog and its activation seems to be exclusive to resting T lymphocytes [3, 4]. IFI16 similarly has only been shown to be activated in a specific population of quiescent, lymphoid-resident human CD4^+^ T cells that are refractory to productive HIV-1 infection [6, 26], and although a mouse orthologue exists, there is little evidence of IFI204 forming a functional inflammasome with caspase-1. NLRP3 is a promising candidate. Not only did we measure significant upregulation of NLRP3 transcripts in Flica-positive cells by RNA-seq and qRT-PCR, but we also detected mitochondrial dysfunction, specifically mtROS generation and mtDNA release, both of which can be sensed by NLRP3 (Figs. 2E–G, 3, and S5A). RNA-seq pathway analysis further revealed mitochondrial and oxidative stress pathways were significantly upregulated in Flica-positive CD8^+^ T cells relative to the Flica-negative and control cells (Figs. 2C, D and S5C). Seahorse data identified metabolic perturbations in cells with active caspase-1, specifically defective OXPHOS (Fig. 4), and TEM images showed significant changes in mitochondrial morphology reminiscent of damage/dysfunction (Figs. 5F and S7–8). Together, these data support a model whereby oxidative stress in alloreactive T cells promotes increased mtROS generation and ultimately mitochondrial dysfunction and DNA release, culminating in inflammasome activation and the activation of caspase-1. A previous study found that transplant of NLRP3-deficient or caspase-1/11-deficient T cells did not protect mice from GvHD, while ASC deficiency in donor T cells prevented disease [9]. While the authors concluded an inflammasome-independent role for ASC in cytotoxic T cells, functional redundancy has been reported for multiple inflammasomes as well as caspases-1 and -8 [12, 27–31], so it is likely that deletion of one pathway does not prevent activation of another. Our data showing inflammasome-bound caspase-1 (p33) and ASC-speck formation in alloreactive T cells clearly demonstrate the involvement of an inflammasome, although the receptor remains to be identified. Future work should investigate NLRP3 and other inflammasomes that sense mitochondrial damage and cytosolic DNA, such as AIM2, IFI16, and NLRP10 in human alloreactive T cells. Notably, we did not measure active caspase-1 in control T cells or T cells activated with α-CD3/CD28 (Fig. S2), demonstrating the specificity of active caspase-1 to alloreactive T cell populations. If ASC is required for GvHD by enhancing the function of pathogenic T cells [9], then therapeutically targeting the inflammasome receptor and/or caspase-1 may specifically eliminate the alloreactive T cell pool to improve GvHD outcomes without influencing Graft-versus-leukemia responses.

Consistent with our findings, increased expression of *Nlrp3* and *proIL-1β* transcripts, as well as increased cleaved caspase-1 and generation of ROS were previously demonstrated in human CD4^+^ T cells stimulated with α-CD3 and α-CD46 [1]. This work found that activation of a complement-NLRP3 axis regulates CD4^+^ Th1 responses and IFN-γ production [1]. In our RNA-seq dataset, complement cascade pathways were upregulated in the CD8^+^ Flica-positive cells (Fig. S5C). Activation of complement signaling pathways could represent another upstream ligand for inflammasome activation in alloreactive T cells.

In addition to the transcriptional changes in inflammatory genes measured in T cells with ongoing caspase-1 activation, we also observed metabolic reprogramming that parallel metabolic phenotypes measured in inflammatory innate immune cells. Inflammatory macrophages significantly reprogram their metabolism away from OXPHOS and towards aerobic glycolysis, which promotes inflammatory functions such as cytokine secretion and phagocytosis. Effector T cells are generally more metabolically active, upregulating various metabolic pathways to meet their increased energy demands. Alloreactive T cells have been shown to adopt both OXPHOS and aerobic glycolysis during aGvHD onset [24, 32]. When we examined the metabolic state in sorted Flica-positive versus Flica-negative populations of T cells isolated from mice with aGvHD, we found that there was a substantial impairment in OCR production from Flica-positive cells (Fig. 4D). Flica-positive CD8^+^ T cells in particular almost exclusively relied on glycolysis to generate ATP (Fig. 4B). In contrast, the metabolic signature in the Flica-negative populations aligned with what has previously been shown for alloreactive T cells, with general increases in ECAR, OCR, and total ATP production, indicating utilization of both metabolic pathways (Fig. 4B–D). In light of a recent report demonstrating a requirement for glycolysis in T cell alloreactivity and GvHD development [33], therapeutically targeting donor T cells that exhibit high levels of active caspase-1 in addition to high glycolytic activity may selectively eliminate these pathogenic cells to ameliorate disease. Further mechanistic insight into the contribution of inflammasome activation to T cell glycolysis is warranted.

Caspase-1 activation in innate immune cells results in cytokine maturation and/or cell death by pyroptosis. Our data demonstrating activation of caspase-1 (Figs. 1D–G, 5D, 6B, and 7D), morphological hallmarks consistent with inflammatory cell death (Figs. 5B, F and S6), GSDMD processing temporally associated with caspase-1 activation (Fig. 5D), and increased PI and Zombie dye uptake (Figs. 5C, S6E, and 6C–E) in mouse and human T cells in in vitro and in vivo models of GvHD suggest that these cells are pyroptotic, though genetic manipulations to inhibit caspase-1 would be necessary to confirm this. Notably, Zombie dye uptake was highly exclusive to T cells with active caspase-1 (Figs. 5C and 6C), which implies that the fate of caspase-1 activation in alloreactive T cells is cell death. However, caspase-1 activation does not always culminate in pyroptosis. Cytokines can be released from viable innate immune cells [34–36], and some cells are able to repair GSDMD pores and survive [37, 38]. We did not measure significant LDH production from alloreactive T cells in vitro, despite measuring increased Zombie dye uptake and IL-18 production from these cells (Fig. S6D, E). It is therefore conceivable that caspase-1 activation is not always cytotoxic in alloreactive T cells.

We also cannot rule out the possibility that the increased membrane permeability was due to alloreactive cells dying by another form of cell death. Apoptosis is an integral aspect of T cell biology, playing a critical role during T cell development in the thymus and in the periphery where activated T cells undergo AICD. This form of cell death is historically considered to be immunologically silent, in contrast to necrotic cell death pathways, which are immunologically active and inflammatory, because they trigger membrane rupture and cell lysis, leading to the release of DAMPs that amplify immune responses. In conditions where caspase-8 is suppressed, T cells have been shown to die by necroptosis [39–41]. Ferroptosis is a form of iron-dependent cell death that may play a role in T cell function [42, 43]. Human T cells can also undergo pyroptosis downstream of inflammasome and caspase-1 activation [3–6]. Additionally, it is becoming increasingly clear that the different cell death modalities do not necessarily operate in isolation; rather, there is significant crosstalk and co-regulation between the key players that orchestrate each pathway [30, 44–49]. In the context of pathogen infection in innate immune cells, a growing body of evidence suggests that multiple programmed cell death pathways can be activated simultaneously to circumvent pathogen-mediated inhibition of a specific death pathway. This concept has been termed PANoptosis, which describes a phenomenon whereby inflammatory cell death occurs through pyroptosis, apoptosis and necroptosis in the same cell [50]. Even in the absence of infection, T cells may utilize multiple cell death pathways as a fail-safe mechanism, to ensure regulation of T cell activity and maintenance of tolerance and homeostasis. Our data demonstrate that multiple mechanisms of cell death may be activated in concert in alloreactive T cells (Fig. 5), although it is unclear if these cell death pathways are engaged in the same cells or in different cell populations.

Despite the high levels of active caspase-1 and GSDMD cleavage measured in alloreactive T cells from mice with aGvHD, we were not able to measure significant IL-1β production in vitro (Fig. 5E), albeit there are limitations to this experiment. Isolated T cells may not behave the same in vitro, the timing and density of cell culture may have prevented us from measuring ample cytokine secretion, or the levels of cytokines produced are below the limit of detection in our assay. T cells are not thought to be primary producers of IL-1β, and previous studies demonstrating inflammasome/caspase-1 activation in T cells in vitro similarly showed no detectable IL-1β secretion [3, 4]. However, we did measure a moderate increase in IL-18 production from alloreactive T cells (Figs. 5E and S6D). It is possible that the downstream consequence of inflammasome/caspase-1 activation in T cells is functionally different than what occurs in innate immune cells. It should be noted that previous studies have measured increased IL-1β transcript and protein expression downstream of TCR signaling [1, 51]. While we were unable to detect IL-1β release from alloreactive T cells, we also observed upregulation of IL-1β transcripts in Flica-positive CD8^+^ T cells (Fig. 2E, F) and intracellular protein levels by ELISA (Fig. 5E). Together, these studies point to a central paradox where caspase-1 can be activated in mouse and human T cells, is capable of cleaving some caspase-1 substrates like GSDMD and IL-18, but does not drive IL-1β release despite proIL-1β upregulation.

Given the disparities in inflammasome responses between mice and humans, we next wanted to confirm that caspase-1 activation similarly occurred in alloreactive human T cells. We measured caspase-1 activation, as well as PI and Zombie dye uptake, in MLR cultures in vitro (Fig. 6). To assess caspase-1 activation in human T cells in vivo, we utilized a xenotransplantation model of GvHD whereby human PBMCs are engrafted into immune-deficient NSG mice. NSG mice contain cells engineered to express human HLA-A2 class I molecules, so that T cells recognize murine xenoantigens in the context of a foreign MHC and proliferate, resulting in pathogenesis that recapitulates many aspects of aGvHD in humans [25]. By day 10, when mice had lost ~20% of their initial body weight, we measured activation of caspase-1 in engrafted human CD4^+^ and CD8^+^ T cells (Fig. 7). These data confirm that caspase-1 is active in alloreactive human T cells. A previous study evaluated the expression of caspase-1 in LPS-stimulated PBMCs from patients with aGvHD found that caspase-1 activation correlated with disease severity [15], which may provide evidence supporting a role for T cell inflammasome/caspase-1 activation in this disease, although a specific assessment of caspase-1 activity in alloreactive T cell populations in patients is needed. Further insight into the mechanism and consequence of inflammasome/caspase-1 activation in transplanted T cells in GvHD may give rise to new therapeutic targets that specifically eliminate pathological alloreactive cells from patients undergoing hematopoietic stem cell transplantation.

### Reporting summary

Further information on research design is available in the Nature Research Reporting Summary linked to this article.

### Supplementary information

Supplemental figures and legends

Reporting Summary

## Data Availability

The RNA sequencing data have been deposited in the GEO database (GSE240196). Any additional information is available from the lead contact upon request.
