# Supplementary material for: Inflammasome activation occurs in CD4+ and CD8+ T cells during graft-versus-host disease
Source: Cell Death Dis. 2023 Sep 25;14(9):632. doi: 10.1038/s41419-023-06138-8 (PMC10519954; doi:10.1038/s41419-023-06138-8)
Supplement: Supplementary file 1 — Supplemental figures and legends [file 41419_2023_6138_MOESM1_ESM.pdf]

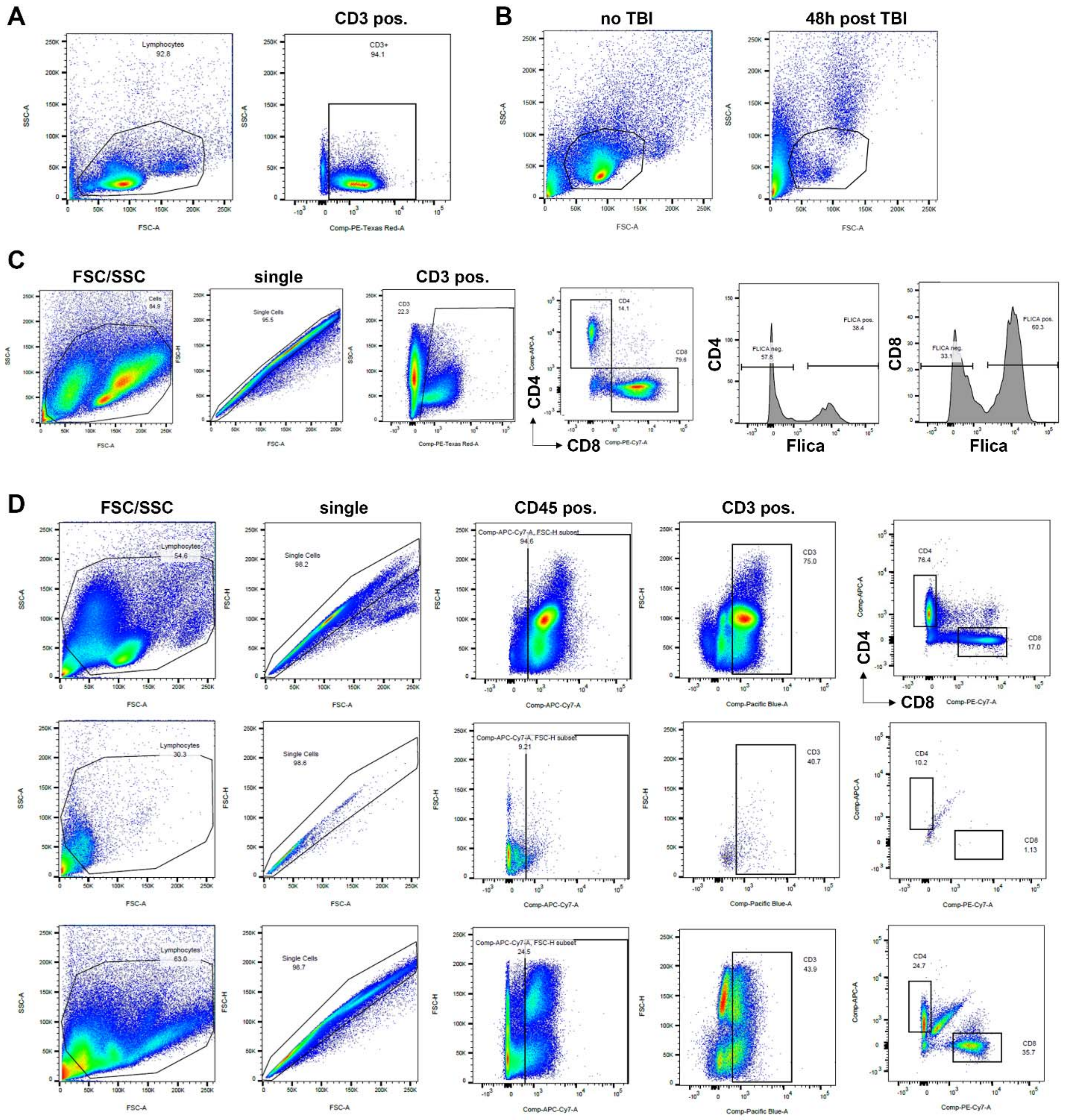

Supplemental Figure 1. Flow cytometric assessment of donor T cell purity, recipient irradiation and gating strategy for T cell populations and caspase-1 activation. (**A**) T cell purity in transferred donor cells was assessed by staining with a-CD3. (**B**) Loss of recipient lymphocytes in the spleen 48h following irradiation of BALB/c mice was measured in animals that received no donor cells. (**C**) Gating strategy for determination of Flica signal in $CD4^+$ and $CD8^+$ T cells from a mouse with aGvHD day 7 following

allogeneic transfer. (**D**) Gating strategy NSG GvHD experiments. Top panels = control day 0 PBMCs, middle panels = splenocytes isolated from irradiation control NSG mice, bottom panels = T cells isolated from splenocytes from NSG mice with aGvHD (day 10).

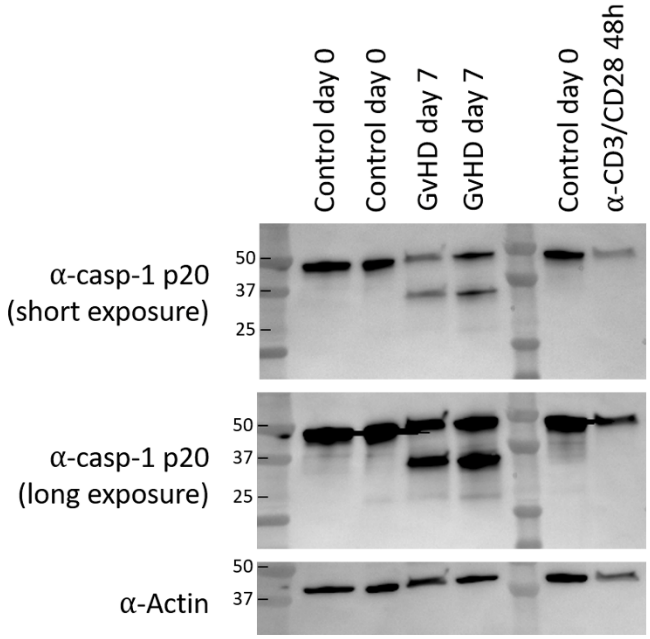

Supplemental Figure 2. **Caspase-1 activation is specific to alloreactive T cells.** Control (day 0) T cells and T cells isolated from mice with aGvHD (day 7) were isolated by positive selection and lysed immediately for western blot analysis. TCR-activated T cells, isolated by negative selection and stimulated with CD3/CD28 Dynabeads for 48h in the presence of IL-2, were lysed for western blot analysis.

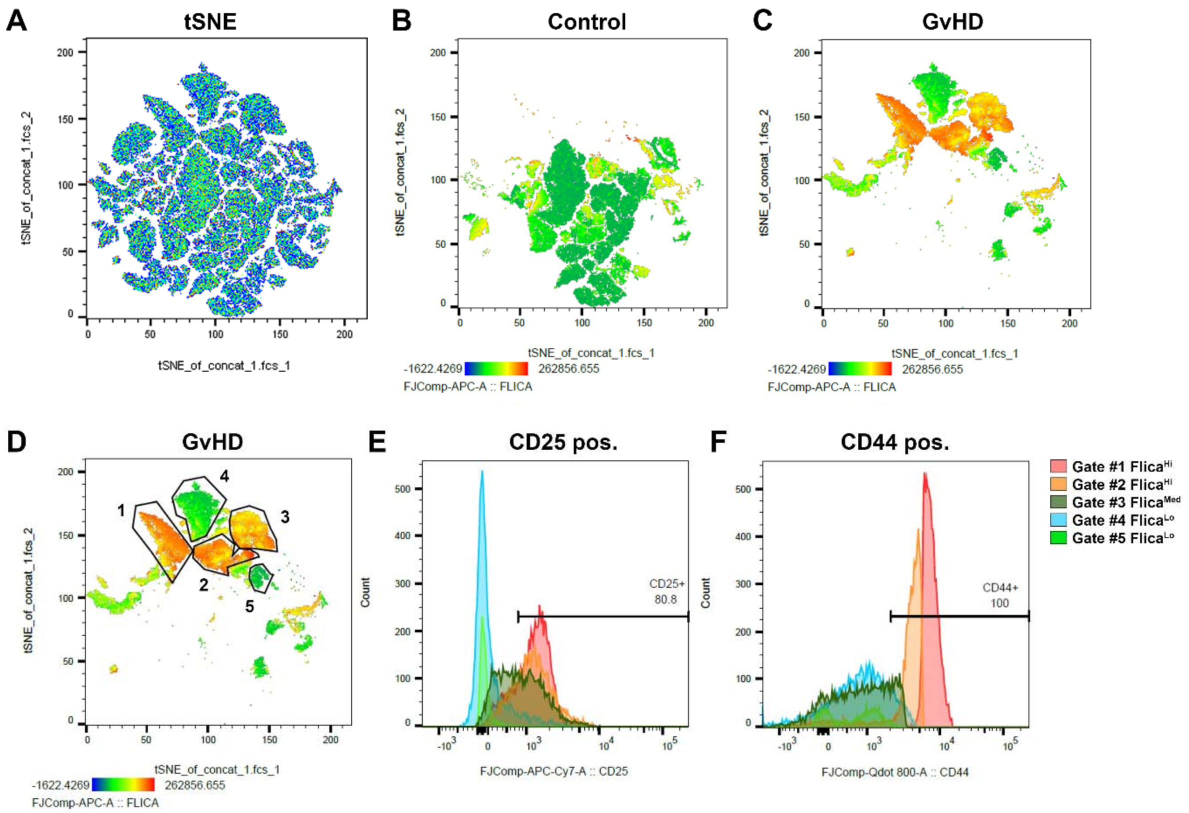

Supplemental Figure 3. t-SNE reveals distinct clusters of alloreactive T cells with active caspase-1. (**A**) Schematic representation of t-SNE plot comparing control and GvHD $CD3^+$ splenocytes. (**B-C**) Control and GvHD tSNE plots were separated and overlaid with a heatmap depicting Flica intensity. (**D-F**) Within the GvHD sample, clusters portraying high, medium or low Flica signal were gated (**D**) and expression of T cell activation markers CD25 (**E**) and CD44 (**F**) were assessed. Depicted are flow plots of splenocytes from n = 1 control and n=1 GvHD mouse, representative from ≥ 3 independent experiments.

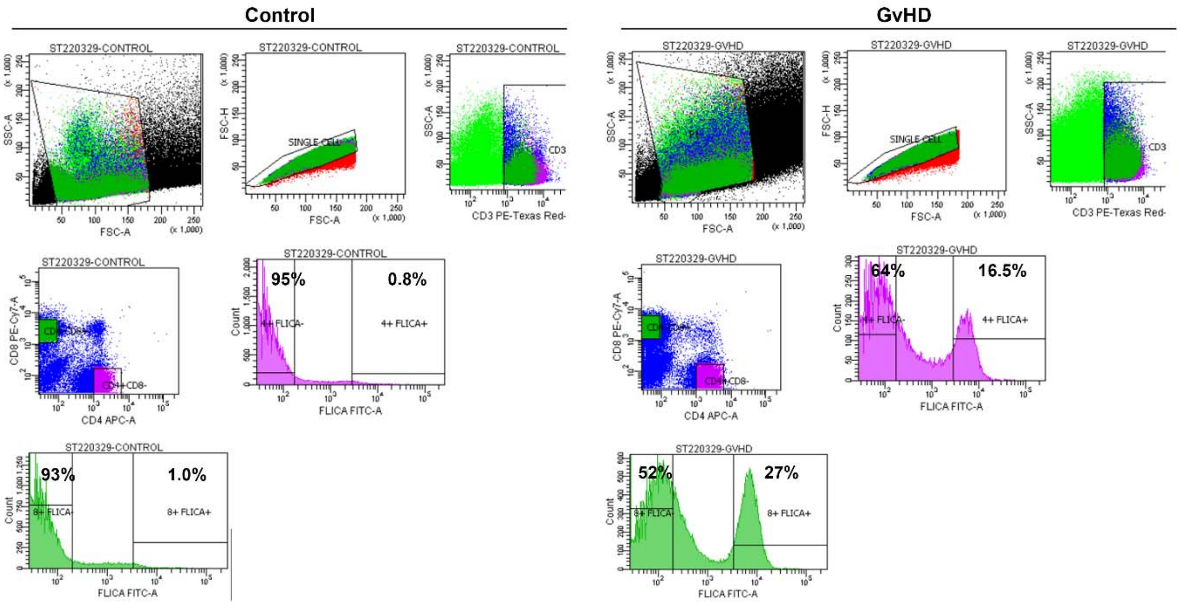

Supplemental Figure 4. FACS strategy to isolate cells with active caspase-1. Splenocytes from 2-3 control C57Bl/6 or 8-10 GvHD mice (day 7 post-transplant) were pooled and stained with the FAM-FLICA Caspase-1 probe followed by antibodies for T cell surface markers, CD3, CD4 and CD8. Shown are raw FACS data from a representative experiment. For each sample, single, $CD3^+$ T cells were sorted into $CD4^+$ or $CD8^+$, Flica-positive and Flica-negative. Cells were collected for further downstream applications.

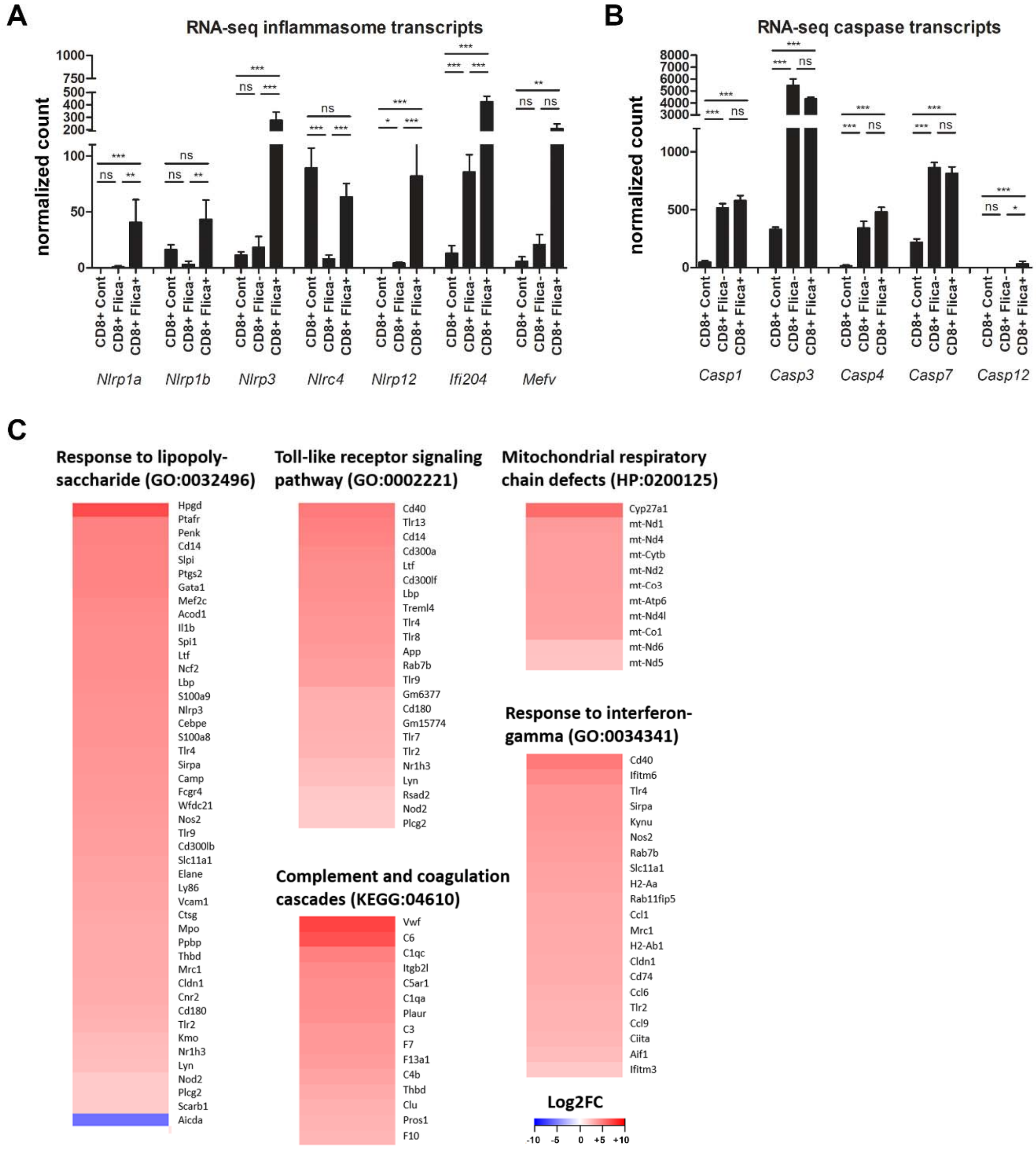

Supplemental Figure 5. Transcriptional changes in inflammasome-forming proteins and caspases, and pathways related to inflammatory and IFN signaling, complement and mitochondrial dysfunction. (**A-B**) Expression of DEGs for transcripts encoding inflammasome-forming proteins and caspases in the indicated cell populations. (**C**) All DEGs within the indicated pathways are shown from RNA-seq datasets comparing the Flica-positive CD8$^+$ T cells to Flica-negative populations.

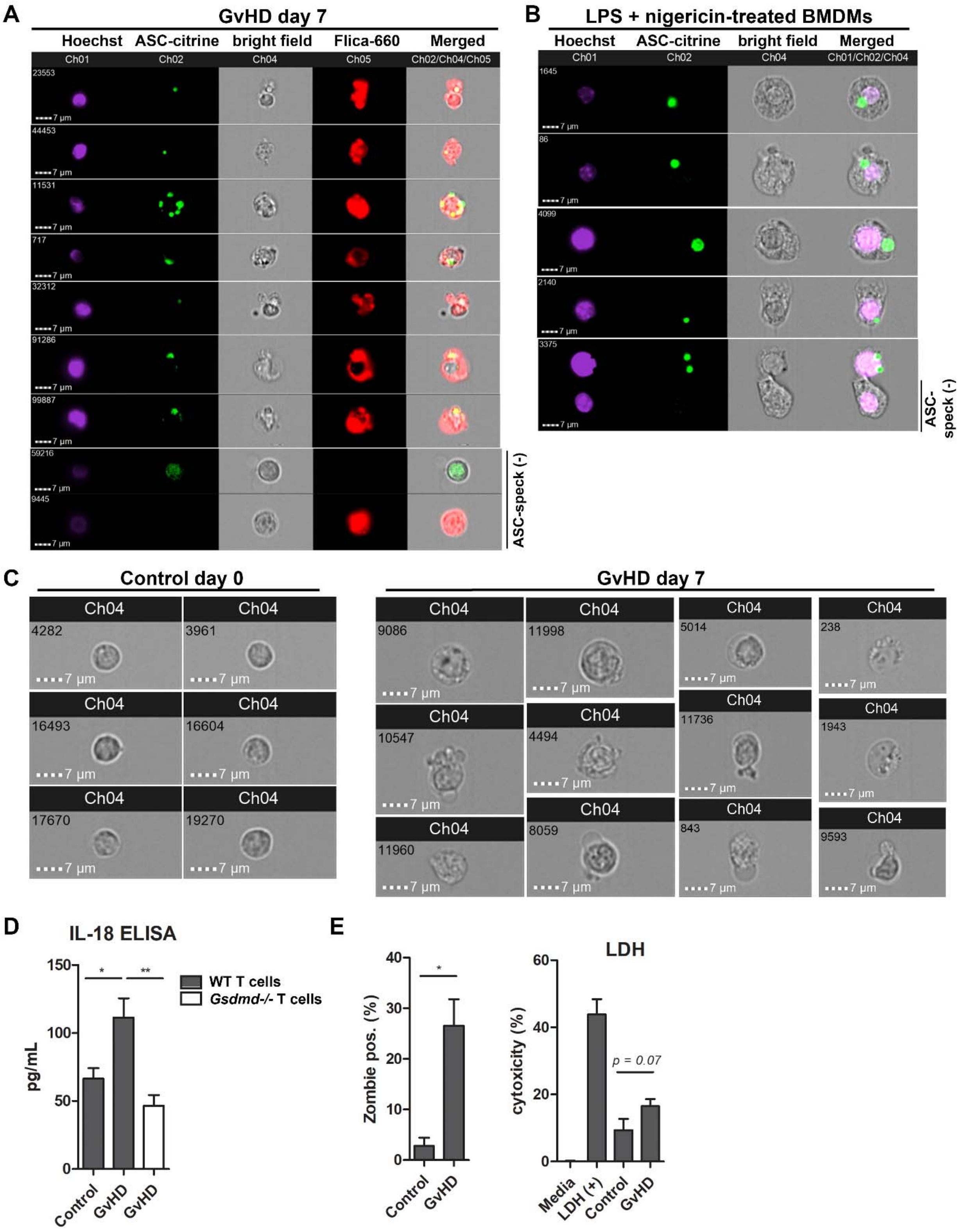
A
GvHD day 7
Hoechst
ASC-citrine
bright field
Flica-660
Merged
ASC-speck (-)
B
LPS + nigericin-treated BMDMs
Hoechst
ASC-citrine
bright field
Merged
ASC-speck (-)
C
Control day 0
GvHD day 7
D
IL-18 ELISA
WT T cells
Gsdmd-/- T cells
pg/mL
Control
GvHD
GvHD
E
Zombie pos. (%)
Control
GvHD
LDH
cytoxicity (%)
p = 0.07
Media
LDH (+)
Control
GvHD

Supplemental Figure 6. ASC speck formation, cytokine production, and cell death in alloreactive T cells. (**A**) Amnis images corresponding to Fig 5B. (**A, top**) Representative images of Flica-positive cells with ASC speck formation (inflammasome activation in donor cell), (**A, bottom**) Flica-negative cells with diffuse ASC-citrine expression (no inflammasome activation in donor cell), or Flica-positive cells with no ASC signal (recipient cell). (**B**) BMDMs derived from ASC-citrine Tg mice were stimulated for 4h with 10 ng/mL LPS followed by 6.5 uM nigericin for 30 minutes. Cells were fixed and stained with Hoechst. Representative Amnis images are depicted. (**C**) ASC-citrine T cells were isolated from control or aGvHD mice (day 7 post-transplant) by negative selection. Representative bright-field images from each population are shown. (**D**) T cells were isolated from mice with aGVHD (day 7 post-transplant of WT or *Gsdmd-/-* T cells) or control mice by negative selection. Cells were cultured *in vitro* for 4h, IL-18 levels were quantified and (**E**) LDH release was measured in the supernatant. Isolated T cells were incubated with Zombie yellow dye for 20 minutes, fixed and analyzed by Flow cytometry. Images shown A-C are representative from 3 experiments. D-E are averages +/- SEM from 3 independent experiments. One-way ANOVA and Bonferroni post hoc test, $p < 0.05$ was considered significant.

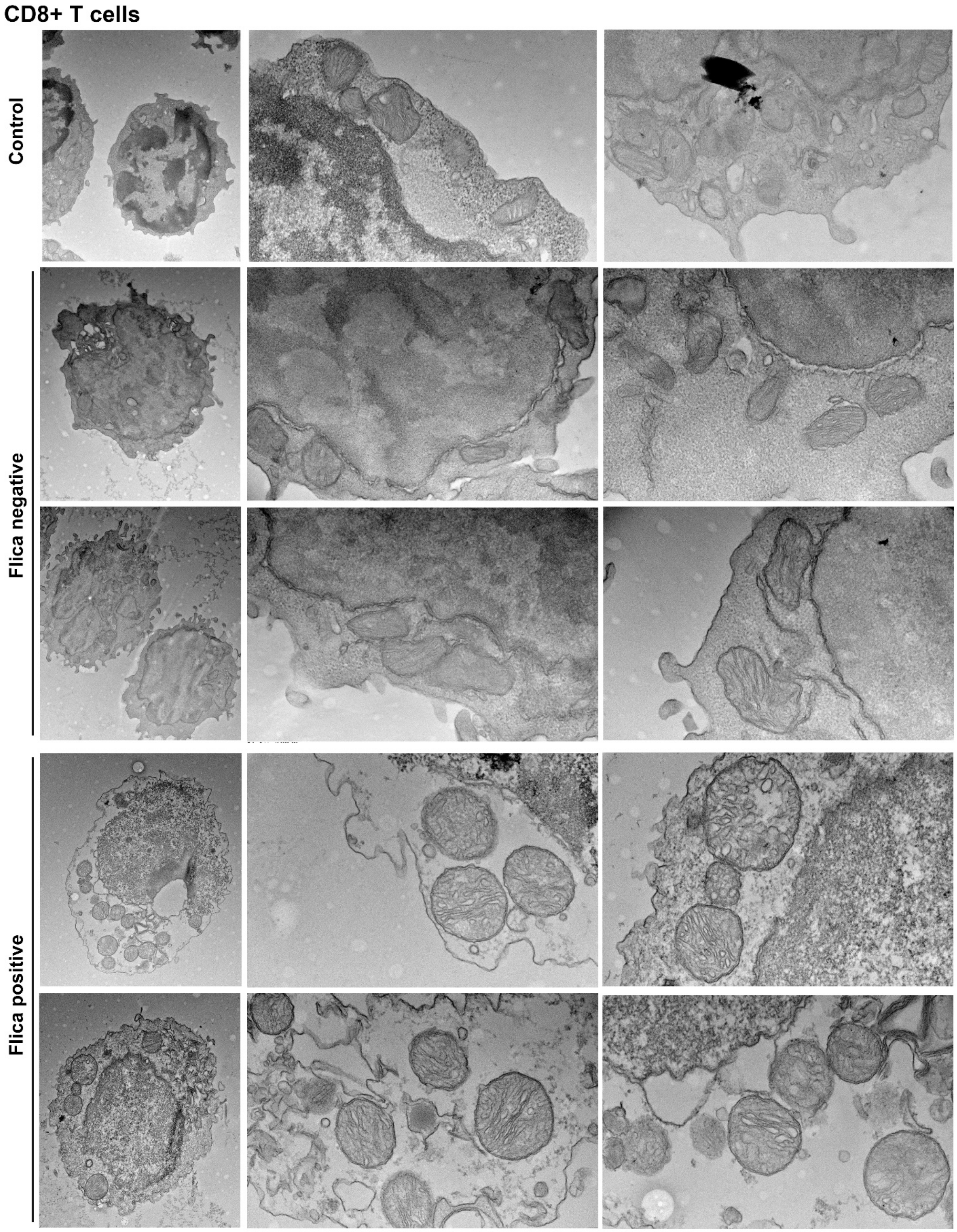

Supplemental Figure 7. Representative TEM images of Flica-positive or Flica-negative CD8^{+} T cells from mice with aGvHD or control mice.

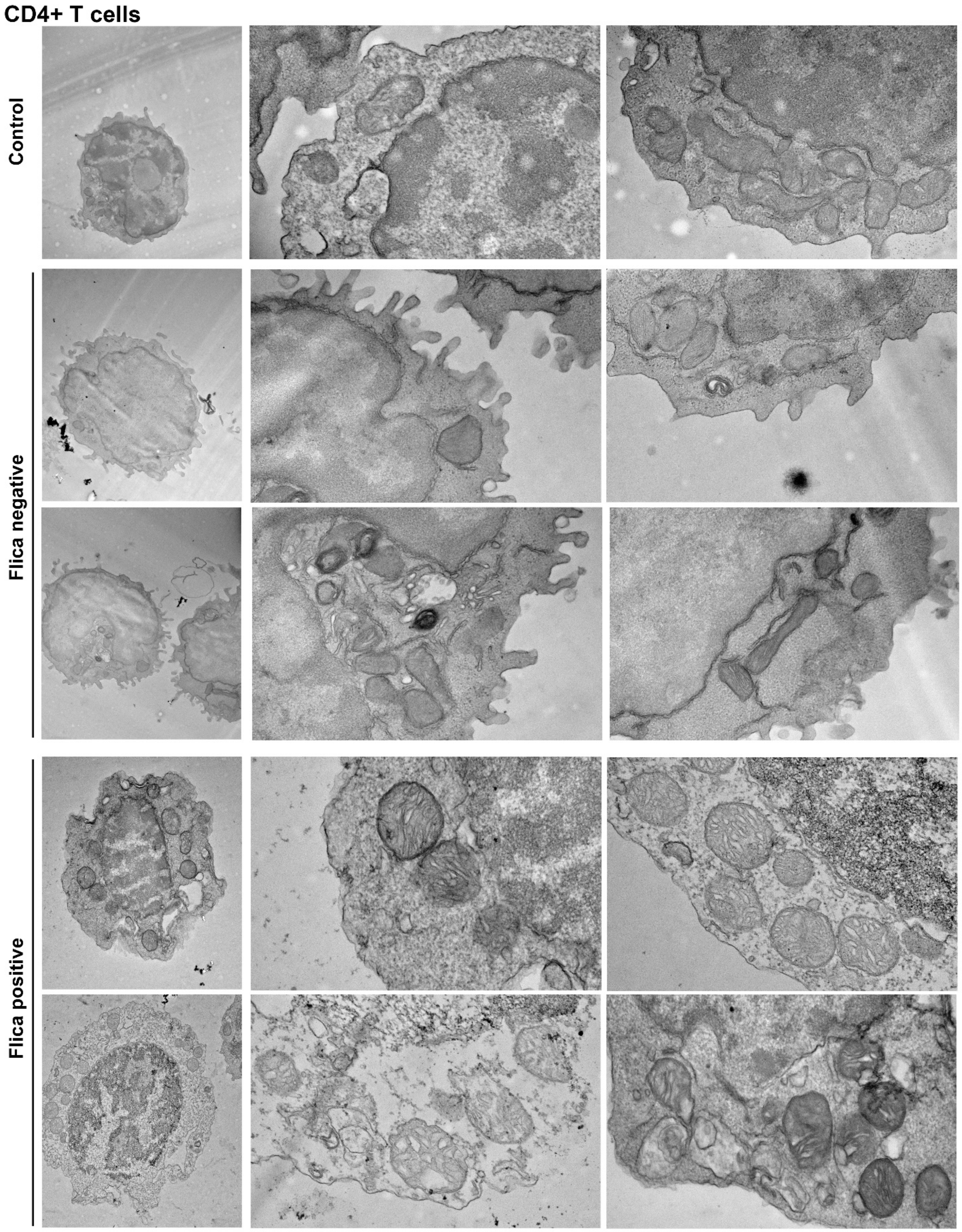

Supplemental Figure 8. Representative TEM images of Flica-positive or Flica-negative CD4$^+$ T cells from mice with aGvHD or control mice.
